# Transparent Conducting Oxides—An Up-To-Date Overview

**DOI:** 10.3390/ma5040661

**Published:** 2012-04-19

**Authors:** Andreas Stadler

**Affiliations:** University of Salzburg, Hellbrunner Str. 34, Salzburg A-5020, Austria; E-Mail: andreas.stadler@sbg.ac.at; Tel.: +43-662-8044-2111; Fax: +43-662-8044-622

**Keywords:** transparent conducting oxide, oxide, TCO, ITO, ZnO:Al, delafossite

## Abstract

Transparent conducting oxides (TCOs) are electrical conductive materials with comparably low absorption of electromagnetic waves within the visible region of the spectrum. They are usually prepared with thin film technologies and used in opto-electrical apparatus such as solar cells, displays, opto-electrical interfaces and circuitries. Here, based on a modern database-system, aspects of up-to-date material selections and applications for transparent conducting oxides are sketched, and references for detailed information are given. As n-type TCOs are of special importance for thin film solar cell production, indium-tin oxide (ITO) and the reasonably priced aluminum-doped zinc oxide (ZnO:Al), are discussed with view on preparation, characterization and special occurrences. For completion, the recently frequently mentioned typical p-type delafossite TCOs are described as well, providing a variety of references, as a detailed discussion is not reasonable within an overview publication.

## 1. Introduction

Transparent conducting oxides (TCOs) are electrical conductive materials with a comparably low absorption of light. They are usually prepared with thin film technologies and used in opto-electrical devices such as solar cells, displays, opto-electrical interfaces and circuitries. Glass fibers are nearly lossless conductors of light, but electrical insulators; silicon and compound semiconductors are wavelength dependent optical resistors (generating mobile electrons), but dopant dependent electrical conductors. Transparent conducting oxides are highly flexible intermediate states with both these characteristics. Their conductivity can be tuned from insulating via semiconducting to conducting as well as their transparency adjusted. As they can be produced as n-type and p-type conductives, they open a wide range of power saving opto-electrical circuitries and technological applications. 

A still valuable overview of transparent conductive oxides is given in [1], basics to material physics of TCOs are discussed in [2], some structural investigation of TCOs was made e.g., in [3], preparation of TCOs was discussed in [4] and substitutes for the most popular transparent conducting oxide, namely ITO (indium-tin oxide), are listed in [5]. Here, based on a modern database-system, aspects of up-to-date material selections and applications for transparent conducting oxides are sketched, and references for detailed information are given. As n-type TCOs are of special importance for thin film solar cell production, ITO and the reasonably priced aluminum-doped zinc oxide (ZnO:Al) are discussed with view on preparation, characterization and special occurrences. For completion, the recently frequently mentioned typical p-type delafossite TCOs are described as well, providing a variety of references, as a detailed discussion is not reasonable within an overview publication.

As transparent conducting oxides are usually compound semiconductors—where the nonmetal part is oxygen—they are discussed along their metal elements. Metals were used as compound materials or dopants (with just a few percent content).

## 2. Transparent Conducting Oxides (TCOs)

### 2.1. TCOs in General

In transparent conducting oxides (TCOs), the nonmetal part, B, consists of oxygen. In combination with different metals or metal-combinations, A, they lead to compound semiconductors, A_y_B_z_, with different opto-electrical characteristics. These opto-electrical characteristics can be changed by doping, A_y_B_z_:D (D = dopant), with metals, metalloids or nonmetals. Hence, metals can be part of the compound semiconductor itself, A, or can be a dopant, D. Scanning the periodic table of elements, with a view on the utilization of metals for TCOs, results in Table 1 (regarding just the 2nd and 3rd period, exclusively aluminum).

**Table 1:** Published results regarding transparent conducting oxide (TCO)-layers, containing metallic elements e.g., from the 2nd and 3rd period of the periodic table of the elements (PE, excluding aluminum), including examples for the later discussed ZnO’s and delafaossites (mayenites)—research with the web of knowledge using “TCO < name of element > oxide”.

Period of the PE	Compound semiconductor	Dopant	Preparation	Characterization	Reference
2	NiO	Li	Pulsed Laser Deposition (different Li-concentr.)	?	[6]
	No TCO-Layers with Be			
3	ZnO	Na, Al	Sol-gel, Annealing	SEM, Photoluminescence	[7,8,9]
	Cr_2_O_3_	Mg, N	Spray Pyrolysis	?	[10]
	CuCrO_2_(Delafossite)	Mg	Sol-gel Technique	?	[11]
	Mg_1−x_Zn_x_O	In	Pulsed Laser Deposition (different substrates)	X-ray diffraction, HRTEM	[12]
	Mg_1−x_Zn_x_O	Al	Radio Frequency Magnetron Sputtering (different substrates)	?	[13]
	Mg_12_Al_14_O_33_ (“Mayenite”)		?	?	[14]
	Al				

Outstanding good optical characteristics have been provided by tin-, indium- and zinc oxides (A = tin, indium, zinc). Well known is, for example, indium tin oxide (ITO), and the doping of zinc oxide with less than 5% aluminum (ZnO:Al). Moreover, doped delafossite and mayenite compounds are of upcoming interest (see Table 1). A variety of preparation and characterization methods was applied to investigate their different chemical structures and physical characteristics. These shall be briefly discussed.

### 2.2. Indium Tin Oxide (ITO)

Indium tin oxide (ITO) is a solid solution of indium(III) oxide (In_2_O_3_) and tin(IV) oxide (SnO_2_), with typically 90%_wt_ In_2_O_3_, 10%_wt_ SnO_2_. It is transparent and colorless as a thin film and yellowish to grey as bulk material. Indium tin oxide is the most widely used transparent conducting oxide (TCO [15]) because of its two key properties, its electrical conductivity and optical transparency. ITO thin films are still deposited with ion assisted plasma evaporation [16], (low temperature) electron beam evaporation [17,18,19], direct current (DC), pulsed DC (PDC), high power pulsed magnetron sputtering (HPPMS), radio frequency (RF) magnetron sputtering [20,21,22,23,24,25], thermal evaporation [25] or pulsed laser deposition (PLD) [26,27,28,29]. Post process thermal annealing steps are discussed for the example in [17,18,19,20], oxygen-plasma treatments in [30] and the influence of acids and bases on ITO thin films in [31]. Investigations were made on electrical [16,17,18,19,20,21,22,23,24,25,26,27,28,30,31], optical [16,17,18,19,20,21,22,23,24,25,26,28,31,32] and structural [17,21,22,26,28,29,32,33] properties of this ternary compound semiconductor. According to structural investigations, the focus was set on the border between amorphous and crystal phases [17] and the growth mechanisms (Volmer-Weber, Frank-van der Merwe) [29]. Band structure and work function are analyzed in [34,35,36].

### 2.3. Aluminum Doped Zinc Oxide (ZnO:Al)

Transparent conducting, aluminum doped zinc oxide thin films (Al_x_Zn_y_O_z_, ZnO:Al) [37,38] contain about 2%_wt_ aluminum and can be produced with spray pyrolysis [39,40,41,42,43,44], sol gel technology [45,46,47,48,49,50,51], electro deposition [52,53], vapor phase deposition [54,55], magnetron DC sputtering [56,57,58,59,60], magnetron RF sputtering [61,62,63,64] or a combination of both the sputter deposition methods [65,66,67,68,69,70,71,72,73,74,75,76,77,78,79,80,81,82]. Moreover, high quality deposition methods using thermal plasmas [83,84], (low pressure (LP), metal organic (MO), plasma enhanced (PE)) chemical vapor deposition (CVD) [85,86], electron beam evaporation [87], pulsed laser deposition [88,89,90,91,92,93] and atomic layer deposition [94] can be applied.

The underlying substrate—crystalline, amorphous or organic—may have an influence on the grown structure and the opto-electronic properties of the thin film [95,96,97,98,99], independent of the used deposition method. For example, in the case of solar cell production, an ultra-thin CdS buffer layer is usually the basis for ZnO:Al deposition [100,101]. Even if the substrate is identical, the layer thickness (deposition time, position upon the substrate) itself influences the physical values of the deposited thin film [102]. A variation of the physical values from the grown thin films can also be reached by changing process parameters, as temperature [103] or pressure [104,105], or by additions to the process gas, as oxygen [106] or hydrogen [107].

Commonly, pure zinc oxides [108,109] are n-doped with aluminum [110,111]. Alternatively, n-doping can be done with metals such as copper, Cu, silver, Ag, gallium, Ga, magnesium, Mg, cadmium, Cd, indium, In, tin, Sn, scandium, Sc, yttrium, Y, cobalt, Co, manganese, Mn, chrome, Cr, and boron, B [88,112,113,114,115,116,117,118,119,120]. p-Doping of ZnO is technologically difficult, but apart fom nitrogen, N, phosphorus, P, seems to be an adequate dopant [121,122,123,124,125,126,127,128].

The opto-electronic properties [129] of these TCO thin films can be changed by post process thermal annealing in an inert gas or reactive gas atmosphere [38,130,131,132]. Especially surface and interface states can be influenced [133,134]. The deterioration of ZnO:Al thin films is discussed in [135].

### 2.4. Delafossite and Mayenite Type Transparent Conducting Oxides

Commonly, ITO- and ZnO-based TCO thin films are n-doped, as p-doping has been shown to be technologically more difficult. Fortunately, for delafossite compound semiconductors this is *vice versa*. They typically show TCO properties with semiconducting p-type characteristics. Delafossites, Cu_x_A_y_O_z_, are commonly ternary material combinations of copper, Cu, one (or more) further metal(s), A, (aboriginal iron, A = Fe) and oxygen, O.

Copper may be replaced by silver [136,137,138,139,140,141], palladium [139] or platinum [142]. As further metal, A, iron [143,144,145], cobalt [138] or chrome [146,147,148,149,150] (without doping hardly transparent) may be used as well as elements of the 2nd group of the periodic table of the elements—strontium [151,152,153,154], barium [155]—or the 3rd group—aluminum [149,156,157,158,159,160,161,162,163,164,165,166,167,168,169], gallium [168,169], indium [170], scandium [171,172], yttrium [173,174,175,176], lanthanum [175,176]. Moreover, other lanthanides such as praseodymium, neodymium samarium and europium have been applied [175,176,177], in order to get ternary semiconductor compounds.

Quaternary semiconductors as for example the Sb-based CuA_2/3_Sb_1/3_O_2_ (A = Mn, Co, Ni, Zn, Mg), respectively AgA_2/3_Sb_1/3_O_2_ (A = Ni, Zn) [138,140] or the Cr-based CuCr_1−x_A_x_O_2_ (A = Mg, Ca, Al) delafossites have been investigated [147,178].

Ag-Cu and Rh-Mg replacements were for example studied in the quinternary structure Cu_1−x_Ag_x_Rh_1−y_Mg_y_O_2_ [179]. 

Oxygen off-stoichiometry, Cu_x_A_y_O_2+d_, has been examined [175,180]. Oxy-sulphide delafossite type TCOs, Cu_x_A_y_O_z_S_α_, were sputtered (CuLa_1−x_OS:Sr_x_, x = 0%–5% [181]) or already existing delafossite-oxide films, Cu_2_In_2_O_5_, sulfurized to CuInS_2_, by annealing in H_2_S [182].

Delafossites have been grown from a melt by a slow cooling-method in air [166,183]. They were deposited using low temperature hydro/solvothermal processes [159,168,184], the sol-gel technology [146,147,149,153,185] and the spray pyrolysis technique [148,158]. Moreover, advanced methods such as (direct current (DC), radio frequency (RF)) magnetron sputtering of prefabricated targets [143,144,156,157,162,164,167,173,181,186], with varying temperature, pressure, oxygen flow or sputter energies [144,161,165], pulsed laser deposition [136,152,163,169,187,188], with varying temperature and pressure [187], thermal evaporation [174], e-beam evaporation technique [154], and (low-pressure (LP), metal-organic (MO)) chemical vapor deposition (CVD) [150] were applied.

Annealing in N_2_, O_2_, air [157,161,162,165] or argon [149] was examined, showing for example a reduction in CuO resp. spinel CuCr_2_O_4_ fraction and formation of highly crystalline films with single-phase delafossite CuCrO_2_ structure [148,164].

The CuA^III^O_2_ group shows increasing band gap from A^III^ = Al, Ga, to In. The largest gap CuInO_2_can be doped both n- and p-type but not the smaller gaps CuAlO_2_ and CuGaO_2_ [189]. Therefore, doping CuInO_2_ with Ca results in p-type, doping with Sn in n-type semiconducting TCO thin films [188,190]. Bidirectional doping is possible for CuFeO_2_, too (p-type: Mg, n-type: Sn [191]). In addition, the electronic structure of CuAO_2_ (A = Al, Ga, Y) was discussed in [192,193,194,195,196] and its luminescent properties in [197]. Defect analyses have been made with the screened-hybrid density functional theory [160].

Additional p-doping is usually performed with Ca, Mg or occasionally with K, in order to increase the conductivity resulting in e.g., CuInO_2_:Ca [151,187], Cu_2_In_2_O_5_:Ca [187], CuYO_2_:Ca [173,174], CuCrO_2_:Mg [138,148,198], CuScO_2_:Mg [138,172] or Cu_2_SrO_2_:K [152]. N-type doping of delafossite TCO thin films is normally done with Sn, e.g., CuInO_2_:Sn [188,190] or AgInO_2_:Sn [136]. Further discussion on doping of delafossite TCOs is shown in [199].

Because of the structural anisotropy of the CuAlO_2_-crystal, anisotropic electrical conductivity was detected in [200]. Ohmic contacts between CuInO_2_ and Cu are reported in [170].

The crystal structures and chemistries are by far the best investigated topics in delafossite (semi)conductor research and systematically discussed in [201,193]; the according temperature dependency is shown in [202].

## 3. Further Aspects to Technological Advances of Transparent Conducting Oxides

Reasons for technical advances in transparent conducting oxides are manifold—influencing aspects are: The investigation of adequate novel materials and material-combinations, as for example the first delafossites by Charles Friedel in 1873 (named after the French mineralogist and crystallographer Gabriel Delafosse); an increasing financial support for research according to political decisions, as for example the increased financial support of solar cell investigations and therefore of TCOs by the present nuclear power phase-out in Germany; the publication of new results, as research groups in industrial companies often reserve important information; and the efficiency of modern literature data-bases, as only included literature can be found and selected.

**Figure 1:**
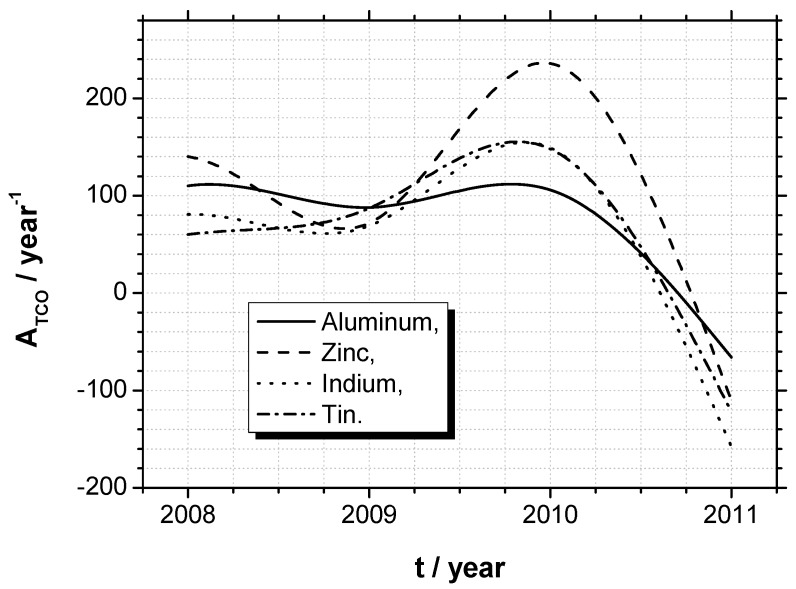
Demonstration of the technical advances in transparent conducting oxides, using the gradient of citations of publications over the years 2007 until November 2011.

Therefore, technical advances in transparent conducting oxides may be illustrated researching the web of knowledge (Thomson Reuters). Applying e.g., the search item “TCO < name of element > oxide” leads to the carefully selected citation statistics, shown in Table 2. Again, the already discussed elements aluminum (Al), zinc (Zn), indium (In) and tin (Sn) show the by far highest nominal citation impacts. In order to demonstrate the technical advances in transparent conducting oxides, the gradient of citations over the years 2007 until 2011 shall be printed for these four elements in Figure 1. This indicates, that the focus of investigation was preferably set on ITO and that A_TCO_ rises until 2010 by about 100 a year. Until 2011, the number of citations per year decreases—not only because this literature research was done in November 2011.

(1) $$A_{TCO}=\frac{\partial citation}{\partial year}$$

**Table 2:** Carefully selected citation report results for TCO-materials, containing metallic elements from the 2nd to the 7th period of the periodic table of the elements (PE)—researched with the web of knowledge using “TCO < name of element > oxide”.

Topic	Citation report						Av. Citations/Year
	2007	2008	2009	2010	2011	Total	
2nd Period							
TCO Li oxide	4	0	3	7	5	19	3.17
TCO Be oxide	x	x	x	x	x	x	x
3rd Period							
TCO Na oxide	0	0	0	0	3	3	3
TCO Mg oxide	8	7	8	8	9	40	8
TCO Al oxide	196	306	394	500	434	2122	192.91
4th Period							
TCO K oxide	1	2	5	3	1	12	2.4
TCO Ca oxide	5	11	5	8	5	47	5.88
Subgroup							
TCO Sc oxide	x	x	x	x	x	x	x
TCO Ti oxide	1	5	14	50	38	114	14.25
TCO V oxide	0	1	9	1	3	18	2
TCO Cr oxide	3	2	2	1	12	28	3.5
TCO Mn oxide	0	0	3	1	1	5	1.25
TCO Fe oxide	x	x	x	x	x	x	x
TCO Co oxide	0	12	23	23	17	75	18.75
TCO Ni oxide	0	0	0	2	5	7	3.5
TCO Cu oxide	18	40	44	73	76	268	33.5
TCO Zn oxide	275	415	487	723	612	3142	184.82
TCO Ga oxide	0	1	15	54	37	107	26.75
5th Period							
TCO Rb oxide	x	x	x	x	x	x	x
TCO Sr oxide	2	7	3	6	1	22	3.14
Subgroup							
TCO Y oxide	0	0	2	1	1	4	1
TCO Zr oxide	0	0	0	1	4	5	2.5
TCO Nb oxide	2	4	8	44	45	103	20.6
TCO Mo oxide	1	17	24	35	21	98	19.6
TCO Tc oxide							radioactive!
TCO Ru oxide	3	8	13	8	1	36	6
TCO Rh oxide	x	x	x	x	x	x	x
TCO Pd oxide	x	x	x	x	x	x	x
TCO Ag oxide	16	43	57	95	67	328	18.22
TCO Cd oxide	37	48	54	119	59	509	36.36
TCO In oxide	247	328	397	546	388	2511	156.94
TCO Sn oxide	346	406	493	641	519	3755	197.63
6th Period							
TCO Cs oxide	x	x	x	x	x	x	x
TCO Ba oxide	x	x	x	x	x	x	x
Subgroup							
TCO Hf oxide	x	x	x	x	x	x	x
TCO Ta oxide	7	8	9	19	10	60	8.57
TCO W oxide	3	5	5	10	8	34	5.67
TCO Re oxide	x	x	x	x	x	x	x
TCO Os oxide	x	x	x	x	x	x	x
TCO Ir oxide	x	x	x	x	x	x	x
TCO Pt oxide	1	0	0	0	1	2	0.4
TCO Au oxide	x	x	x	x	x	x	x
TCO Hg oxide	3	4	9	5	3	24	4.8
TCO Tl oxide	x	x	x	x	x	x	x
TCO Pb oxide	x	x	x	x	x	x	x
TCO Bi oxide	x	x	x	x	x	x	x
Lanthanide Series							
TCO La oxide	0	0	2	0	1	3	1
TCO Ce oxide	0	0	1	1	0	39	2.17
TCO Pr oxide	x	x	x	x	x	x	x
TCO Nd oxide	x	x	x	x	x	x	x
TCO Pm oxide	x	x	x	x	x	x	x
TCO Sm oxide	0	0	1	10	8	19	6.33
TCO Eu oxide	0	0	1	8	5	14	4.67
TCO Gd oxide	0	0	0	1	4	5	2.5
TCO Tb oxide	x	x	x	x	x	x	x
TCO Dy oxide	0	0	0	9	6	15	7.5
TCO Ho oxide	x	x	x	x	x	x	x
TCO Er oxide	x	x	x	x	x	x	x
TCO Tm oxide	x	x	x	x	x	x	x
TCO Yb oxide	x	x	x	x	x	x	x
TCO Lu oxide	x	x	x	x	x	x	x
7th Period							
TCO Fr oxide	x	x	x	x	x	x	x
TCO Ra oxide	x	x	x	x	x	x	x
Actinide Series							
TCO Ac oxide	x	x	x	x	x	x	x
TCO Th oxide	x	x	x	x	x	x	x
TCO Pa oxide	x	x	x	x	x	x	x
TCO U oxide							radioactive!
…							radioactive!

**Figure 2:**
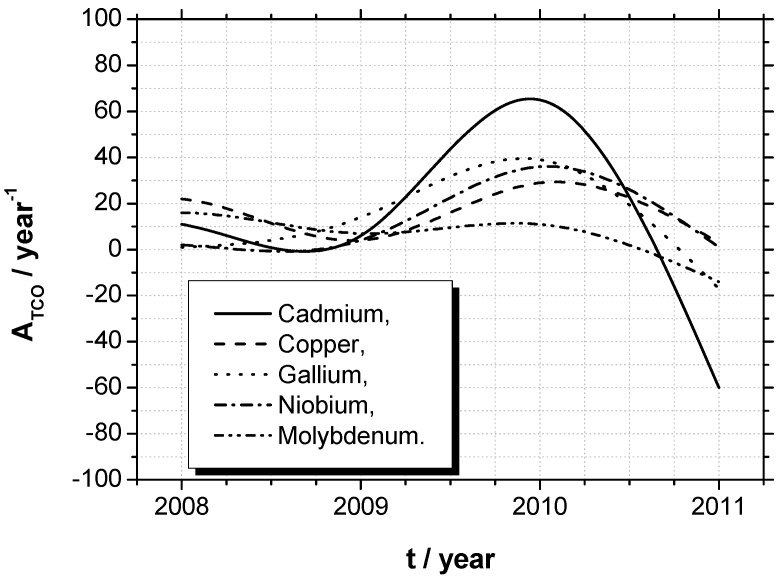
Demonstration of the technical advances in transparent conducting oxides, using the gradient of citations of publications over the years 2007 until November 2011.

Despite these four elements, let us regard the next five metals, which exhibit the most average citations per year in TCO-related publications, see Table 2, Figure 2. Hence, Cadmium (Cd) is discussed as CdO:D (D = Ga, Sn, Sm, Eu, Gd, or Dy), CdIn_2_O_4_ or Cd_2_SnO_4_, where H_2_-annealing is frequently applied to widen the energy gap [203,204,205].

Copper (Cu) represents the group of doped and undoped CuO_2_ and delafossites, see above.

Gallium (Ga) on the one hand is used as dopant, D (about 2%_at_), for ZnO and CdO. On the other hand Ga is the metallic part, A, of Ga_2_O_3_. Based on this, gallium zinc oxide (GZO: ZnGa_2_O_4_) is produced with 90%_wt_ of Ga_2_O_3_ and 10%_wt_ of ZnO. Moreover, aluminum gallium zinc oxide (AGZO) is a combination of aluminum zinc oxide (AZO) and GZO, respectively indium gallium zinc oxide (IGZO) a combination of IZO and GZO [206,207].

Niobium (Nb) is exclusively used as dopant, with an atomic concentration of about 3%_at_–6%_at_, primarily for TiO_2_:Nb but also for SnO_2_:Nb [208,209].

Molybdenum (Mo) is usually used in comparatively high conductive TCOs. Mo is a dopant for ZnO (MZO) or In_2_O_3_ (IMO). MoO is also applied in layer stacks with silver, Ag [210,211,212].

The upcoming importance of transparent conductive materials for thin film solar cells, opto-electrical interfaces, displays and opto-electrical circuitry widens the area of investigation. So, exotic dopants, such as sodium (Na) [213] and manganese (Mn) [214] for zinc oxides (ZnO), zirconium (Zr) [215], platinum (Pt) and tungsten (W) [216] for indium oxide (In_2_O_3_), ITO and IGZO or lanthanum (La) [217] for strontium stannate La_x_Sr_1−x_SnO_3_ have been discussed in the last few years.

Finally, ultra-thin metals without any oxygen content (except natural oxidation in air at room temperature)—as for example nickel (Ni)—have been applied as optical transparent conducting materials [218].

## 4. Conclusions

Based on a modern database-system, aspects of up-to-date material selections and applications for transparent conducting oxides have been sketched; references for detailed information have been given for the interested reader. As n-type TCOs are of special importance for thin film solar cell production, indium-tin oxide (ITO) and the reasonably priced aluminum-doped zinc oxide (ZnO:Al) have been discussed with view on preparation, characterization and special occurrences. For completion, typical p-type delafossite TCOs have been described the same way, providing a variety of references, as a detailed discussion is not reasonable within an overview-publication. Moreover, absolutely unusual, novel TCO materials have been discussed and their presence and development in the world of science pointed out. Trends have been shown.

As transparent conducting oxides are usually compound semiconductors—where the nonmetal part is oxygen—they have been discussed along their metal elements. Metals were used as compound materials or dopants (with just a few percent content).
